# First Morphological Description of Larvae of Two Endemic Goby Species from Chinese Waters

**DOI:** 10.3390/ani15243570

**Published:** 2025-12-11

**Authors:** Shouhai Liu, Yuwen Zeng, Ying He, Biqi Zheng, Haisheng He, Lijia Liu, Cheng Zhang, Weimin Yao, Junsheng Zhong

**Affiliations:** 1 Shanghai Universities Key Laboratory of Marine Animal Taxonomy and Evolution, Shanghai Ocean University, Shanghai 201306, China; liushouhai1986@163.com (S.L.); m240300989@st.shou.edu.cn (Y.Z.); 2 Key Laboratory of Marine Ecological Monitoring and Restoration Technologies, Ministry of Natural Resources, Shanghai 201206, China; heying13218360888@163.com (Y.H.); zhengbiqi@ecs.mnr.gov.cn (B.Z.); hehaisheng2018@163.com (H.H.); liulijia@ecs.mnr.gov.cn (L.L.); 13230301295@163.com (C.Z.); 3 East China Sea Ecology Center, Ministry of Natural Resources, Shanghai 201206, China; 4 Engineering Technology Research Center of Marine Ranching, Shanghai Ocean University, Shanghai 201306, China; 5 Ningde Marine Center, Ministry of Natural Resources, Ningde 352100, China

**Keywords:** *Amoya chusanensis*, *Wuhanlinigobius polylepis*, ichthyoplankton, ontogenetic development, larval identification

## Abstract

*Amoya chusanensis* and *Wuhanlinigobius polylepis* are endemic goby species (Gobiidae, Perciformes) in Chinese waters, with *A. chusanensis* occurring in the East China Sea and *W. polylepis* distributed in waters off Shanghai and Hainan. We applied mitochondrial COI gene sequencing to larval specimens collected from the Yangtze River estuary in August 2017, confirming species identity for both endemic gobies. This study presents the first detailed morphological descriptions of their larvae, supported by high-resolution photographic documentation, and compares their ontogenetic development with related gobiid species. Comparative analysis revealed that the melanin pigmentation pattern along the ventral body margin—extending from the anus to the posterior terminus of the notochord—exhibits species-specific characteristics and serves as a diagnostic criterion for larval identification. These findings establish a novel approach for early developmental research on endemic Chinese fishes and provide scientific basis for conservation strategies targeting their nursery grounds.

## Simple Summary

Two small fish species, *Amoya chusanensis* and *Wuhanlinigobius polylepis*, live only in Chinese coastal waters and nowhere else in the world. These bottom-dwelling fish inhabit shallow areas where rivers meet the sea. Because they exist in such limited areas, they are especially vulnerable to environmental changes and human activities. We collected young fish from the Yangtze River estuary and used genetic testing combined with detailed microscopic examination to confirm their identities and describe what they look like during early life stages for the first time. We discovered that the pattern of dark spots along the belly of these young fish differs between species and can be used to tell them apart from related species. Understanding where these fish spawn and raise their young is essential for protecting them. Our findings provide important information for conservation efforts, especially as the estuary environment changes due to rising water temperatures, altered river flow from dams, and fishing activities. This research establishes methods for studying other rare Chinese fish species and helps guide policies to protect critical nursery areas where these endemic fish grow.

## 1. Introduction

*Amoya chusanensis* is a small benthic fish species inhabiting nearshore areas, found in brackish and freshwater environments or shallow coastal waters, distributed exclusively along the East China Sea coast [1,2,3]. *Wuhanlinigobius polylepis* is a small warm-temperate benthic fish species inhabiting estuarine brackish and freshwater areas, distributed in Shanghai, Fujian, Taiwan, and Hainan [4,5]. Both *A. chusanensis* and *W. polylepis* are endemic species to Chinese seas [1,2,3,4,6]. Endemic species are defined as those whose distribution is restricted to a specific geographical region or continent due to historical, ecological, or physiological factors, and which do not occur elsewhere [7]. Recent studies indicate that the current rate of species loss is 1000 times faster than the natural extinction rate and one million times faster than the rate of species formation [8]. Due to their restricted distributions, endemic species are particularly vulnerable to environmental changes and human activities, making species conservation a critical challenge for these taxa. China is one of the world’s most biodiverse countries, but simultaneously faces severe threats to its biodiversity, including many endemic Chinese species [9,10]. While terrestrial flora and fauna and freshwater fish endemics have been extensively studied [11,12,13,14,15,16,17], research on the early development of marine endemic species has progressed slowly due to sampling difficulties and the complexity of species identification. This knowledge gap is particularly pronounced compared to terrestrial and freshwater endemic species research [18].

Species identification forms the foundation for developing conservation strategies. This study employs an integrated approach combining morphological analysis with DNA barcoding [19,20] to provide the first descriptive records of larval-stage characteristics of the Chinese endemic gobies *A. chusanensis* and *W. polylepis*. The larval stage represents a critical phase in fish life cycles, and research on larval morphological characteristics and distribution patterns helps reveal the ecological features of spawning and nursery grounds, providing scientific evidence for population dynamics monitoring and conservation strategy development for endemic species.

## 2. Materials and Methods

### 2.1. Sample Collection and Morphological Identification

Samples were collected during ecological surveys conducted in the Yangtze River estuary and adjacent waters in August 2017. Following the Marine Survey Specification (GB/T12763.6-2007 [21]), ichthyoplankton sampling was performed using both vertical and horizontal tows with shallow-water Type I nets (net length: 145 cm, mouth diameter: 50 cm, mouth area: 0.5 m^2^, mesh size: 0.505 mm) and large zooplankton nets (net length: 280 cm, mouth diameter: 80 cm, mouth area: 0.5 m^2^, mesh size: 0.505 mm). Specimens were preliminarily processed in the field and preserved in absolute ethanol.

In the laboratory, samples were examined and preliminarily identified using a Nikon SMZ 25 stereomicroscope (Nikon, Tokyo, Japan) based on larval morphological characteristics [19,22,23,24,25]. Photographs were taken, and morphometric parameters including body length, head length, eye diameter, and pre-anal length were measured using NIS-Elements D v5.01 software.

### 2.2. DNA Barcoding Analysis

Approximately 100 mg of muscle tissue was sampled for total DNA extraction using a Marine Animal Tissue Genomic DNA Extraction Kit (Tiangen Biotech Co., Ltd., Beijing, China). The mitochondrial COI gene fragment (approximately 650 bp) was amplified using universal primers F1: 5′-TCRACYAAYCAYAAAGAYATYGGCAC-3′ and R1: 5′-TAGACTTCWGGGTGRCCRAAGAATCA-3′.

PCR amplification was performed in a total reaction volume of 50 μL containing 10× PCR buffer (5 μL), dNTP (4 μL at 2.5 mmol/L), forward and reverse primers (2 μL each at 10 mmol/L), Taq DNA polymerase (0.8 μL at 5 U/μL), template DNA (1 μL), and double-distilled water to reach the final volume of 50 μL.

Amplification was conducted using an AG-22331 PCR systemV2.12 (Eppendorf, Hamburg, Germany) with the following thermal cycling conditions: initial denaturation at 94 °C for 5 min; 35 cycles of 94 °C for 30 s, 52 °C for 45 s, and 72 °C for 1 min; final extension at 72 °C for 10 min; and storage at 4 °C. PCR products were detected by 1.0% agarose gel electrophoresis, then subjected to gel recovery and bidirectional sequencing by Shanghai Jieli Biotechnology Co., Ltd. (Shanghai, China), followed by sequence assembly.

### 2.3. Data Processing

Sequences obtained from sequencing and those downloaded from GenBank were proofread using the DNAStar software v7.1package. All sequences were aligned and sorted using Clustal X v1.83, with redundant columns at both ends removed. Valid COI gene sequences were subjected to BLAST analysis in the Bold System (Barcode of Life Data System) database and GenBank for species identification.

Sequences were aligned and edited in MEGA v7.0 software. Phylogenetic trees were constructed using the Maximum Likelihood (ML) method with the General Time Reversible + G + I model. Bootstrap confidence values for each branch were calculated through 1000 replications using the Bootstrap method.

## 3. Results

### 3.1. Systematic Classification

Family Gobiidae
  Subfamily Gobiinae
    Genus *Amoya* Herre, 1927
      *Amoya chusanensis* (Herre, 1940)
        Genus *Wuhanlinigobius* Huang, Zeehan & Chen, 2013
          *Wuhanlinigobius polylepis* Wu et Ni, 1985

### 3.2. Molecular Analysis

**Figure 1:**
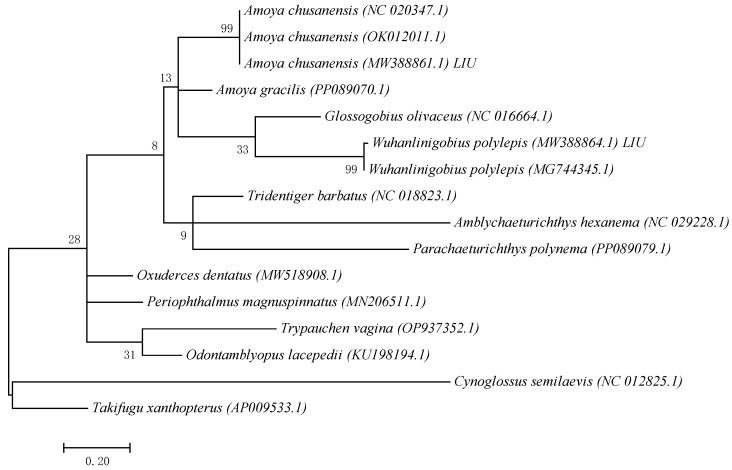
ML phylogenetic tree constructed based on mtCOI gene fragment sequences.

Based on COI sequences of 11 gobiid species obtained from GenBank and the COI sequence fragments of 2 gobiid species obtained in this study, a Maximum Likelihood (ML) phylogenetic tree was constructed using *Cynoglossus semilaevis* (Cynoglossidae) and *Takifugu xanthopterus* (Tetraodontidae) as outgroups (Figure 1). The results showed that the samples clustered with *A. chusanensis* and *W. polylepis*, respectively. The sequences of *A. chusanensis* (MW388861.1) and *W. polylepis* (MW388864.1) were simultaneously uploaded to the GenBank database.

### 3.3. Morphology

#### 3.3.1. *Amoya chusanensis* (Herre, 1940)

**Juvenile stage.** The body measured 12.67 mm in length, with a body depth of 17% of the total length, a head length of 29% of the total length, and an eye diameter of 26% of the head length. The anus was located at the center of the body. The body was slightly transparent, with the head slightly elevated. The eyes were large. The mouth cleft was relatively small, with the base of the mouth cleft positioned slightly anterior and ventral to the eye center.

**Pigmentation.** The dorsal surface of the head exhibited scattered small punctate melanophore clusters. The dorsal margin of the body showed melanophores arranged in a linear pattern. The ventral margin from the anus to the base of the anal fin at the end of the notochord bears two symmetrical rows of linearly arranged punctate melanophore bands, each row containing seven melanophores. A large stellate melanophore cluster was present at the midpoint between the anus and the end of the notochord. From the large punctate melanophore cluster to the end of notochord, there was one linearly arranged punctate melanophore band. Two larger melanophore patches were present at the caudal fin base: one circular and one bar-shaped. Melanophores were visible on the swim bladder within the body cavity.

#### 3.3.2. *Wuhanlinigobius polylepis* Wu et Ni, 1985

Larval samples of *W. polylepis* were divided into two groups (body lengths 6.16 mm and 7.18 mm), representing early and late post-flexion stages, respectively. Morphological differences between stages were mainly reflected in body depth and eye diameter proportions.

**Post-flexion stage.** The body measured 6.16 mm in length, with a body depth of 16% of the total length, a head length of 24% of the total length, and an eye diameter of 21% of the head length. The anus was located at 52% of the body length from the anterior end. The body was relatively transparent. The head was flattened without an obvious elevation. The eyes were moderately sized and nearly circular. The mouth cleft was relatively small, with the lower jaw longer than the upper jaw, and the base of the mouth cleft positioned slightly anterior and ventral to the eye center. Melanophores were visible on the swim bladder within the body cavity.

**Meristics:** There were 11 second dorsal fin rays, 7 anal fin rays, and 17 caudal fin rays, with the pelvic fins membranous.

**Pigmentation:** The ventral margin from the anus to the base of the anal fin at the end of the notochord bears two symmetrical rows of linearly arranged punctate melanophore bands, each row containing eight melanophores. A large clustered melanophore patch was present at the midpoint between the anus and the end of the notochord. From the clustered melanophore patch to the end of the notochord, there is one linearly arranged punctate melanophore band.

**Post-flexion stage (late)**. The body measured 7.18 mm in length, with a body depth of 19% of the total length, a head length of 24% of the total length, and an eye diameter of 18% of the head length. The anus was located at 52% of the body length from the anterior end. The melanophores were visible on the swim bladder within the body cavity, with the pelvic fins membranous.

**Pigmentation:** The ventral margin from the anus to the base of the anal fin at the end of the notochord bears two symmetrical rows of linearly arranged punctate melanophore bands, each row containing eight melanophores. A large clustered melanophore patch was present at the midpoint between the anus and the end of the notochord. From the clustered melanophore patch to the end of the notochord, there was one linearly arranged punctate melanophore band.

### 3.4. Comparative Study of Gobies

Based on the results of this study and previously reported morphological characteristics of several goby species [23], *W. polylepis*, *A. chusanensis*, *Acentrogobius pflaumii*, *Parachaeturichthys polynema*, and *Cryptocentrus filifer* exhibit differences in the presence or absence of dorsal pigmentation, and the number and arrangement patterns of pigmentation at the anal fin base. The taxonomic key points are as follows:
1 (2) Dorsal pigmentation present on the body*Amoya chusanensis*.2 (1) Dorsal pigmentation absent on the body.
3 (6) Pigmentation at the anal fin base arranged symmetrically and orderly on both sides.
4 (5) One large pigment spot present directly posterior to the anal fin base*Wuhanlinigobius Polylepis*.5 (4) No large pigment spot directly posterior to the anal fin base*Cryptocentrus filifer*.6 (3) Pigmentation at the anal fin base arranged asymmetrically.
7 (8) Black pigment vesicles well-developed on the ventral surface of the caudal peduncle*Parachaeturichthys polynema*.8 (7) Black pigment vesicles poorly developed on the ventral surface of the caudal peduncle*Acentrogobius pflaumii*.

## 4. Discussion

The integration of morphological characteristics with DNA barcoding represents a valuable complement to traditional morphological approaches for understanding early developmental changes in fish species [20]. Traditional taxonomic identification methods are often limited by the substantial morphological differences across the three developmental stages—eggs, larvae, and juveniles [26]—or by the high morphological similarity between different species, making species-level identification challenging [27]. The dramatic morphological remodeling and high interspecific convergence make species identification and precise developmental stage classification based solely on traditional morphological features (such as pigmentation patterns, fin fold differentiation timing, and myomere counts) particularly challenging. This is especially problematic for morphologically similar sympatric sister species or cryptic species complexes, where misidentification risks are significant [28,29,30]. The application of DNA barcoding technology effectively addresses these issues and improves the accuracy of taxonomic identification for fish eggs, larvae, and juveniles [31,32]. Morphological descriptions based on this foundation have enhanced the taxonomic identification resources for ichthyoplankton and established a diagnostic framework for accumulating taxonomic data on early developmental stages of fish [19,20,33].

**Figure 2:**
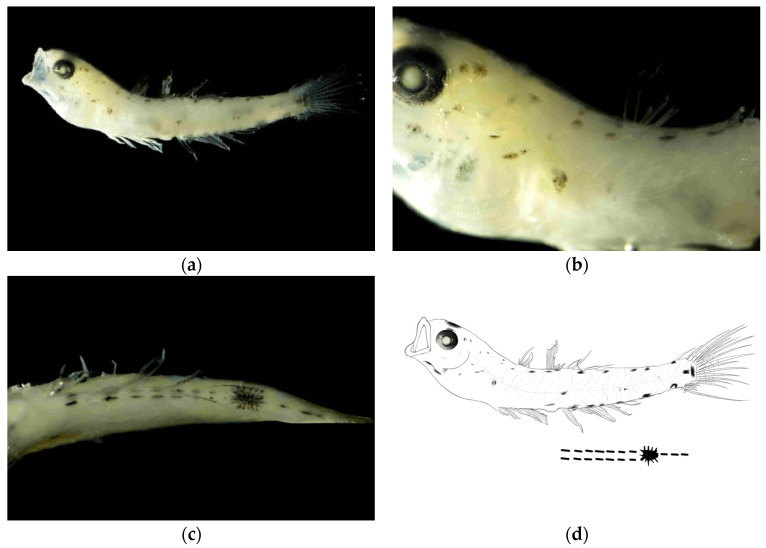
Larval morphology of *Amoya chusanensis* at juvenile stage (body length 12.67 mm). (**a**) Lateral view showing overall body morphology and pigmentation pattern. (**b**) Enlarged view of head region showing eye position, mouth cleft orientation, and dorsal melanophore distribution. (**c**) Enlarged view of ventral body margin showing the characteristic symmetrical arrangement of melanophores from anus to caudal fin base, with the distinctive large stellate melanophore cluster at the midpoint and two prominent melanophore patches at the caudal fin base. (**d**) Line drawings of juvenile stage.

**Figure 3:**
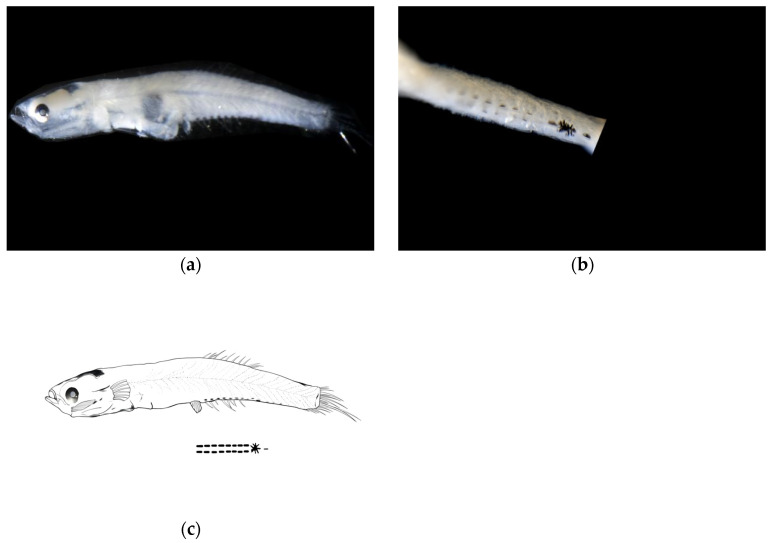
Larval morphology of *Wuhanlinigobius polylepis* at early post-flexion stage (body length 6.16 mm). (**a**) Lateral view showing overall body morphology, flattened head profile, and characteristic ventral pigmentation pattern. (**b**) Enlarged view of ventral body region illustrating the symmetrical arrangement of melanophores from anus to caudal fin base, featuring the distinctive large clustered melanophore patch at the midpoint and the linearly arranged punctate melanophore band extending posteriorly. (**c**) Line drawings of post-flexion stage.

**Figure 4:**
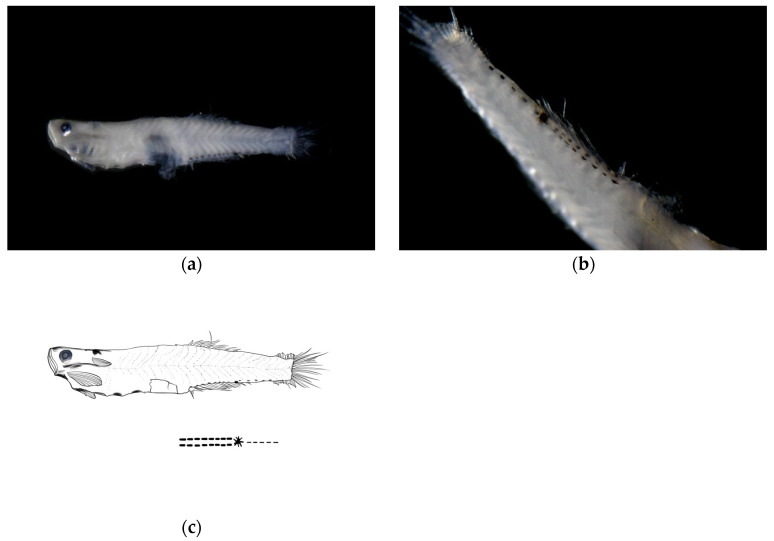
Larval morphology of *Wuhanlinigobius polylepis* at late post-flexion stage (body length 7.18 mm). (**a**) Lateral view showing overall body morphology with increased body depth compared to early post-flexion stage, and consistent ventral pigmentation pattern. (**b**) Enlarged view of ventral body region demonstrating the maintained symmetrical arrangement of melanophores from anus to caudal fin base, with the characteristic large clustered melanophore patch at the midpoint and posteriorly extending punctate melanophore band. (**c**) Line drawings of post-flexion stage.

**Figure 5:**
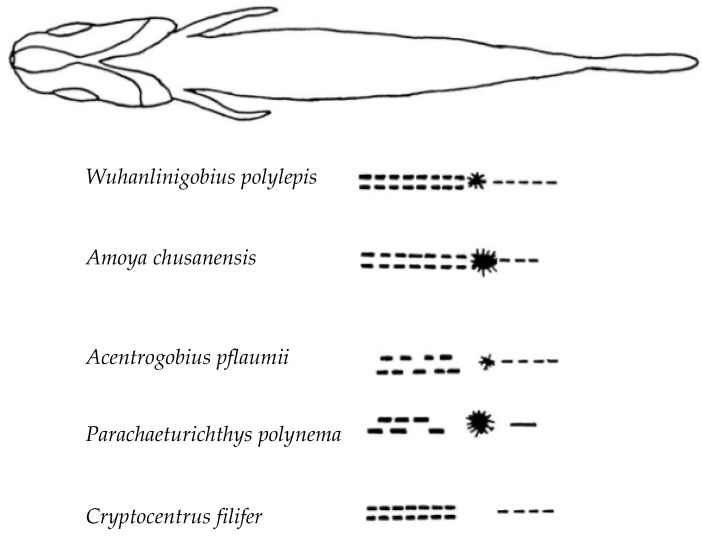
Comparative morphology of five goby species larvae showing diagnostic pigmentation patterns. Lateral views illustrating key taxonomic features: presence/absence of dorsal pigmentation, symmetrical versus asymmetrical arrangement of melanophores at the anal fin base, and the distinctive large pigment spot posterior to the anal fin base in *Wuhanlinigobius polylepis*, enabling species-level identification during larval stages.

Pigmentation serves as a crucial characteristic for distinguishing larval and juvenile fish species [20,22,23]. The distribution patterns of pigmentation (including head, body sides, fin ray bases, and ventral regions), density, and the timing and morphology of specific patches (such as post-orbital spots, caudal fin base spots, and dorsal fin base melanophores) provide key species identification clues during the larval and juvenile stages of gobies [34,35]. In this study, *A. chusanensis* exhibited extensive pigmentation distributed on the dorsal head region, dorsal body margin, and ventral body margin (Figure 2), while *W. polylepis* showed pigmentation only along the ventral body margin (Figure 3 and Figure 4). Previous reports indicate that the pigmentation patterns along the ventral body margin of goby larvae and juveniles are highly diverse [23,36,37,38]. The ventral body margin pigmentation in *A. pflaumii*, *P. polynema*, and *C. filifer* larvae and juveniles primarily consists of linear melanophores arranged in two rows [23], which is similar to that observed in *A. chusanensis* and *W. polylepis* (Figure 5). The pigmentation distribution characteristics along the ventral body margin of *A. chusanensis* and *W. polylepis*, particularly the post-anal pigmentation patterns, serve as important distinguishing features from other goby species.

*Amoya chusanensis* is endemic to China, distributed along the East China Sea coast [1,2,3]. *Wuhanlinigobius polylepis* has a slightly broader distribution than *A. chusanensis*, occurring in Shanghai, Fujian, Taiwan, and Hainan, and is also endemic to China [4,5]. *W. polylepis* has been listed in the China Species Red List (2004) [3]. Both endemic species, *A. chusanensis* and *W. polylepis*, are small benthic fish inhabiting brackish waters, belonging to the Gobiidae family with similar ecological niches [3]. Studies on the early development of *A. chusanensis* and *W. polylepis* can enhance our understanding of the spawning and nursery grounds of these endemic fish species in Chinese seas. Combined with data on rising seawater temperatures (0.5–1.0 °C) [39,40] and changing salinity gradients in the Yangtze River estuary over the past decade, monitoring the distribution dynamics, spawning ground changes, and key life history characteristics of *A. chusanensis* and *W. polylepis* larvae can provide evidence for assessing the impacts of the Three Gorges Project and fishing moratorium policies on estuarine ecosystems. This has important implications for future watershed–estuary integrated management and adaptive conservation strategy development, including optimizing reservoir operations, dynamically adjusting fishing restrictions, and establishing climate-resilient protected areas.

## 5. Conclusions

This study presents the first comprehensive morphological descriptions of larval stages for two Chinese endemic goby species, *Amoya chusanensis* and *Wuhanlinigobius polylepis*, combining traditional morphological analysis with DNA barcoding technology. The integration of mitochondrial COI gene sequencing with detailed photographic documentation successfully confirmed species identities and revealed distinctive ontogenetic characteristics. Comparative analysis demonstrated that the melanin pigmentation pattern along the ventral body margin, particularly from the anus to the posterior terminus of the notochord, provides reliable diagnostic criteria for distinguishing these endemic species from morphologically similar gobiid larvae. The presence of dorsal pigmentation in *A. chusanensis* and the symmetrical arrangement of ventral melanophores with a characteristic large pigment spot in *W. polylepis* serve as key identification features that enhance the taxonomic framework for early developmental stages of Chinese endemic fishes.

The documentation of larval *A. chusanensis* and *W. polylepis* in the Yangtze River estuary confirms this region as an important nursery ground for these endemic species, providing critical baseline data for conservation strategy development. As both species are restricted to Chinese waters and face increasing environmental pressures from climate change, anthropogenic activities, and hydrological alterations, understanding their early life history and distribution patterns becomes essential for effective population monitoring and habitat protection. This research establishes a methodological foundation for future ichthyoplankton surveys targeting endemic species and provides scientific evidence to support integrated watershed–estuary management policies, including the optimization of fishing moratorium periods and the designation of climate-resilient marine protected areas to safeguard critical spawning and nursery grounds.

## Data Availability

The data supporting the findings of this study are available from the corresponding author, Weimin Yao (ywm@ecs.mnr.gov.cn), upon reasonable request.
